# Evaluation of lateral flow immunochromatographic assay for diagnostic accuracy of c*ryptococcosis*

**DOI:** 10.1186/s12879-020-05368-x

**Published:** 2020-09-04

**Authors:** Li-Min Xie, Geng-Ling Lin, Hao-Neng Dong, Ying-Xia Liao, Ye-Ling Liu, Jian-Feng Qin, Xu-Guang Guo

**Affiliations:** 1 Department of Clinical Medicine, The Third Clinical School of Guangzhou Medical University, Guangzhou, 511436 China; 2 Department of Clinical Laboratory Medicine, The Third Affiliated Hospital of Guangzhou Medical University, Guangzhou, 510150 China; 3 Department of Clinical Pharmacy, The Pharmic School of Guangzhou Medical University, Guangzhou, 511436 China; 4 Key Laboratory for Major Obstetric Diseases of Guangdong Province, The Third Affiliated Hospital of Guangzhou Medical University, Guangzhou, 510150 China; 5 Key Laboratory of Reproduction and Genetics of Guangdong Higher Education Institutes, The Third Affiliated Hospital of Guangzhou Medical University, Guangzhou, 510150 China

**Keywords:** Lateral flow immunochromatographic assay, Lateral flow assay, Cryptococcosis, Diagnostic

## Abstract

**Background:**

*Cryptococcus* is a conditional pathogenic fungus causing cryptococcosis, which is one of the most serious fungal diseases faced by humans. Lateral flow immunochromatographic assay (LFA) is successfully applied to the rapid detection of cryptococcal antigens.

**Methods:**

Studies were retrieved systematically from the Embase, PubMed, Web of Science, and Cochrane Library before July 2019. The quality of the studies was assessed by Review Manager 5.0 based on the Quality Assessment of Diagnostic Accuracy Study guidelines. The extracted data from the included studies were analyzed by Meta-DiSc 1.4. Stata 12.0 software was used to detect the publication bias.

**Results:**

A total of 15 articles with 31 fourfold tables were adopted by inclusion and exclusion criteria. The merged sensitivity and specificity in serum were 0.98 and 0.98, respectively, and those in the cerebrospinal fluid were 0.99 and 0.99, respectively.

**Conclusions:**

Compared to the urine and other samples, LFA in serum and cerebrospinal fluid is favorable evidence for the diagnosis of cryptococcosis with high specificity and sensitivity.

## Background

Cryptococcosis is mainly caused by *Cryptococcus*, an opportunistic pathogen. *Cryptococcus* genus is based on *C. neoformans*, *C. deneoformans*, *C. gattii*, and other non-pathogenic. Those strains of serotype A or var. grubii are considered to be *C. neoformans* and serotype D or *var. Neoformans* are considered to be *C. deneoformans*. The strains of *C. gattii* consist of five species: *C. gattii*, *C. bacillisporus*, *C. deuterogattii*, *C. tetragattii* and *C. decagatti i *[1]. *C. gattii* and *C. neoformans* are responsible for almost all cryptococcal infections in humans [2]. Besides, people with low immunity have a high probability of being infected with *Cryptococcus*, for example, hunman immunodeficiency virus (HIV) patients and patients with long-term use of glucocorticoids, immunosuppressants, broad-spectrum antibiotics, and anti-tumor drugs [3, 4]. All organs of humans can be infected with *Cryptococcus*. Without complement and anti-*Cryptococcus* growth factors in cerebrospinal fluid (CSF), cryptococcal meningitis (CM) is the main clinical manifestation of the cryptococcal infection in the central nervous system [5]. In 2014, the number of cryptococcal antigen-positive people worldwide was 278,000, and the global incidence of cryptococcal meningitis was 223,100. Additionally, annual global deaths from cryptococcal meningitis were estimated at 181,100 and 135,900 deaths in sub-Saharan Africa and 15% of AIDS-related deaths are caused by cryptococcal meningitis worldwide [6]. Thus, cryptococcosis has become a serious global public health problem.

However, the cryptococcal infection is short of specificity with diverse clinical manifestations. Cryptococcosis is frequently misdiagnosed at the early stage [7]. The diagnosis of cryptococcosis relies on the cultivation of conventional fungal and bacterial culture media from biological samples (CSF, sputum and skin biopsies, etc.), cytological examination of centrifuged CSF deposits and histopathological staining of other body fluids, or using of latex agglutination, enzyme immunoassay techniques to detect cryptococcal polysaccharide capsular antigen (CrAg) which has shed in serum and CSF during infection [8]. Molecular methods, although available and extensively used for research purposes, are not used currently in routine clinical practice [7]. These methods have the advantage of high specificity, but the sensitivity is low. Moreover, these tests are time-consuming and require auxiliary equipment [9].

In 2009, Immuno-Mycologics (IMMY) invented a new cryptococcal antigen detection method, lateral flow immunoassay (LFA), for diagnosis of cryptococcal infection. LFA is a rapid diagnostic method for the quantitative or qualitative detection of analytes in complex mixtures providing results within 5–30 min [8]. LFA can detect samples without special auxiliary equipment, which can also be used for the determination of single samples and preserve the results of the test. In addition to IMMY LFA, BIOSYNEX® CryptoPS is a rapid immunochromatographic test for the semi-quantitative detection and titration of *Cryptococcus* capsular antigens in serum, plasma, whole blood and CSF to guide the diagnosis of cryptococcal infections, especially in cases of meningitis. Biosynex CryptoPS can detect the four serotypes of *Cryptococcus*, and provides results within 10 min [10]. In July 2011, the U.S. Food and Drug Administration has approved lateral flow immunoassay (LFA) (Immy, Inc., Norman, OK, USA) as a semi-quantitative tool for the rapid detection of cryptococcal capsular polysaccharide antigen in the serum or CSF [11]. The application of LFA rapid detection of *Cryptococcus* greatly shortens the time for the diagnosis of the disease, and also has a certain positive effect on the subsequent early treatment. Therefore, we collected relevant articles for the meta-analysis to assess LFA for the diagnostic accuracy of cryptococcosis.

## Methods

### Search strategy and source

Four investigators systematically searched all the articles about the *Cryptococcus* and LFA before July 2019 in the Embase, PubMed, Web of Science, and Cochrane Library databases. We used the keywords "*cryptococcus*, torula, filobasidiella" and "lateral flow immunochromatographic assay, LFA, colloidal gold immunochromatography: for advanced search. Geographical restrictions were not applied in these articles.

### Study selection and screening criteria

Two investigators systematically screened all of the articles by pre-established screening criteria. The inclusion criteria were as follows: (1) Studies published in English. (2) The purpose of the study was related to LFA and cryptococcosis. (3) Studies are limited to original research. (4) Studies related to diagnostics. (5) Data can be extracted to construct fourfold tables. The exclusion criteria were as follows: (1) Duplicate studies, abstracts, conference abstracts, case reports, reviews, editorials. (2) Studies without a reference standard or a detailed number of samples. (3) Samples not from humans. (4) LFA as the reference standard.

### Data extraction

In the process of carefully reading the included articles, the investigators simultaneously extracted related data from the studies, including the name of the first author, year of article, study design, geographical distribution of strains, patient population, reference standard, brand of LFA-test, sample type, true positive (TP), false positive (FP), true negative (TN), and false-negative (FN). The process of extracting data is carried out independently by the investigators, and finally, the synthesis results were compared.

### Quality assessment standard

We used the Quality Assessment of Diagnostic Accuracy Study (QUADAS-2) guidelines [12] to assess the quality of included studies. Then, we analyzed the risk of bias and applicability concerns by Review Manager 5.0, including patient selection, reference standard, index test, flow, and timing. If the assessment results conflicted, the investigators reviewed the original studies, and a third investigator would intervene to achieve consensus.

### Statistical analysis

We analyzed the extracted data, such as specificity, sensitivity, negative likelihood ratio (NLR), positive likelihood ratio (PLR), and diagnostic odds ratio (DOR), from the included studies using meta-DiSc 1.4 software. Also, we analyzed the summary receiver operating characteristic (SROC) curve and calculated the area under the curve (AUC). According to the sample types, these studies were analyzed by different methods. Due to the lack of adequate data on urine and other samples in the included articles, these samples were analyzed by Review Manager 5.0 software for sensitivity and specificity. Finally, publication bias was evaluated by Stata12.0 software.

## Results

### Search results

**Fig. 1:**
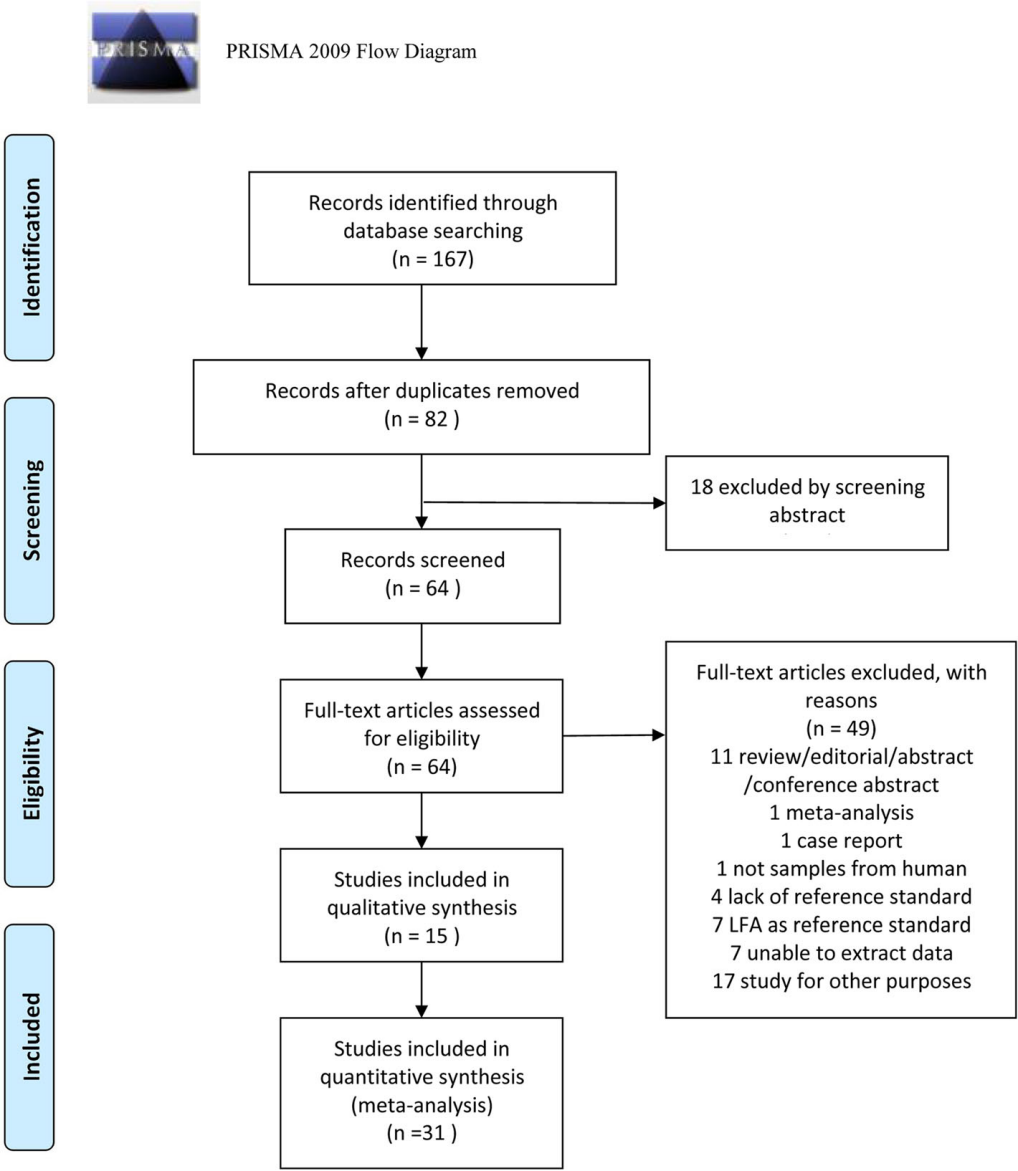
Flow diagram of study identification and inclusion

A total of 167 publications were retrieved, which decreased to 82 after excluding the duplicates. Also, 18 studies were excluded after screening the abstracts. After full-text review, we excluded 49 articles. The reasons for exclusion as shown in Fig. 1. Finally, we included 15 qualified articles [9, 11, 13–25].

### Characteristics of eligible studies

Fifteen studies were published between 2011 and 2019. 13/15 articles reported data from serum samples, seven collected CSF samples, two contained urine samples, and one contained the samples of fingerprick capillary blood and whole venous blood. A total of 9312 samples were included in the meta-analysis, with an average of 620 (range 59–3447) samples. The brands of LFA-tests of total included studies were IMMY. Table 1 summarizes the characteristics of these studies.

**Table 1:** Characteristics of the included studies (*n* = 15)

No.	First author	Year	Geographical distribution of strains	Study design	Patient population	Sample type(s)	Sample size	Reference standard	Brand of LFA-test
1	Lindsley	2011	Thailand	prospective	HIV	Serum Urine	538	EIA	IMMY
2	Binnicker	2012	USA	prospective& retrospective	SC	Serum	634	LA	IMMY
3	McMullan	2012	Australia	retrospective	SC	Serum	106	Comprehensive reference3	IMMY
4	Escandón	2013	Colombia	retrospective	HIV	Serum	421	LA	IMMY
5	Hansen	2013	USA	prospective	SC	Serum CSF	1000	EIA	IMMY
6	Rugemalila	2013	Tanzania	prospective	SC	Serum	319	LA	IMMY
7	Boulware	2014	Uganda& South Africa	prospective& retrospective	HIV SM	CSF	666	Culture	IMMY
8	Lourens	2014	South Africa	prospective	HIV SM	CSF	465	Culture/LA	IMMY
9	Rivet-Dañon	2015	France	prospective& retrospective	IFI1 SC HIV	Serum CSF	292	LA	IMMY
10	Suwantarat	2015	America	retrospective &prospective	SC	Serum CSF	1047	EIA/Enhanced reference4	IMMY
11	Jitmuang	2016	America	retrospective	HIV-N	Serum CSF	59	LA	IMMY
12	Cáceres	2017	Colombia	retrospective	CIB2	Serum CSF	83	LA	IMMY
13	Frola	2017	Argentina	prospective	HIV	Serum	123	Comprehensive reference5	IMMY
14	Temfack	2018	Cameroon	prospective	HIV	Serum	186	EIA	IMMY
15	Drain	2019	South Africa	prospective	HIV	VWB FCB Urine	3447	EIA/Combined reference6	IMMY

*HIV* hunman immunodeficiency virus, *SC* suspected cryptococcosis, *SM* suspected meningitis, *HIV-N* HIV-negative, *CSF* cerebrospinal fluid, *VWB* venous whole blood, *FCB* fingerprick capillary blood, *LA* latex agglutination method, *EIA* enzyme-linked immunoassay, *LFA* lateral flow assay, *IMMY* Immuno-Mycologics. 1:patients proven or probable invasive fungal infection other than cryptococcosis; 2:patients with or without diagnosis of cryptococcosis were randomly selected from a collection of iological samples stored in the CIB’s biobank; 3:Cryptococcosis was proven if the organism was detected by one or more of ulture, histopathology or molecular tests; 4:An enhanced reference method includes data from histopathology, cytopathology, ungal culture, and patient clinical history in addition to EIA results; 5:Pathogen identification of isolates from positive blood cultures was performed using standard microbiology methods (morphological and biochemical tests); 6:A combined reference standard for either a positive CrAg EIA or latex agglutination test

### Quality assessment

**Fig. 2:**
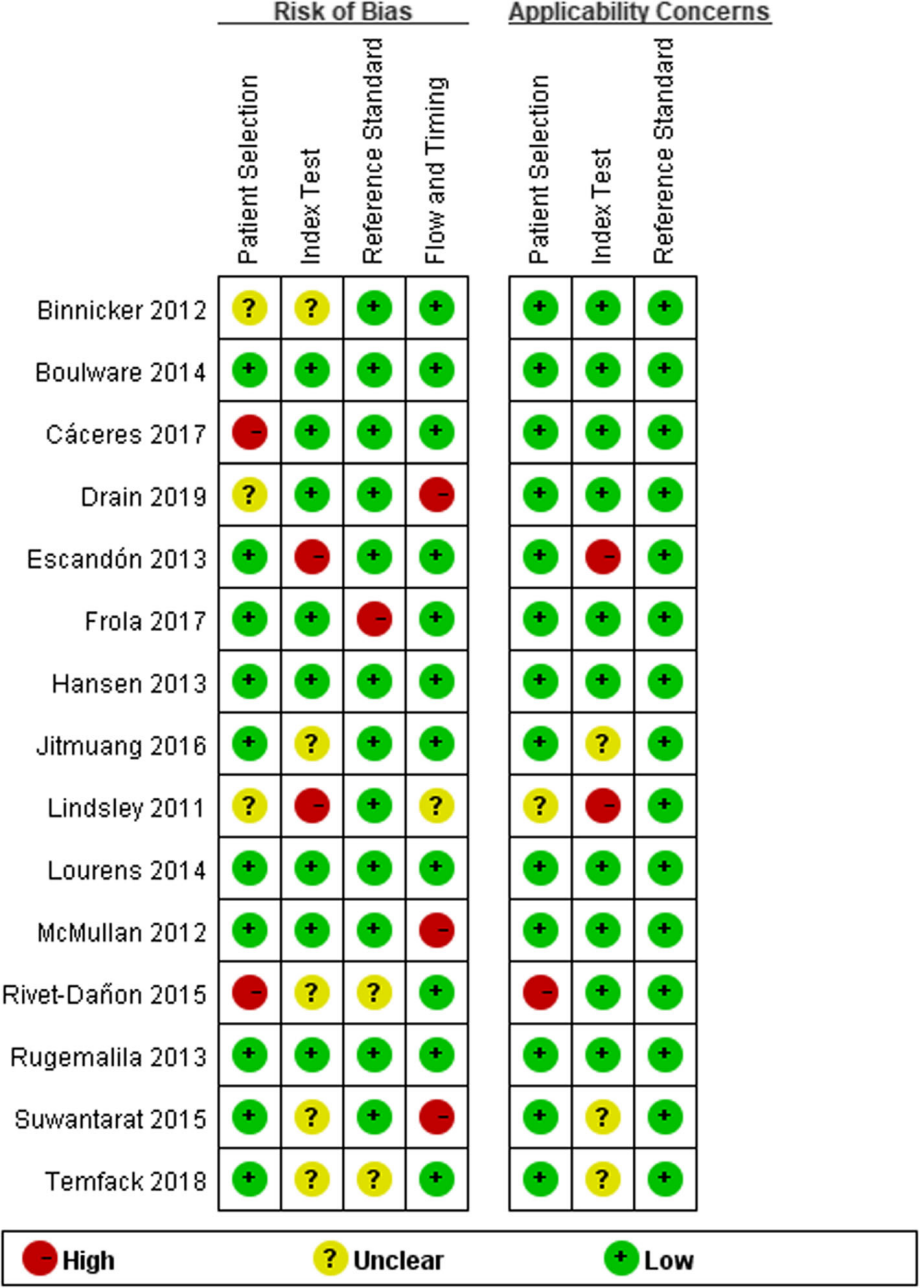
Quality evaluation of the included studies

We assessed the quality of 15 articles using Review Manager 5.3. (Fig. 2).

### Data analysis

We classified the studies into different categories due to the different sample types.

**Fig. 3:**
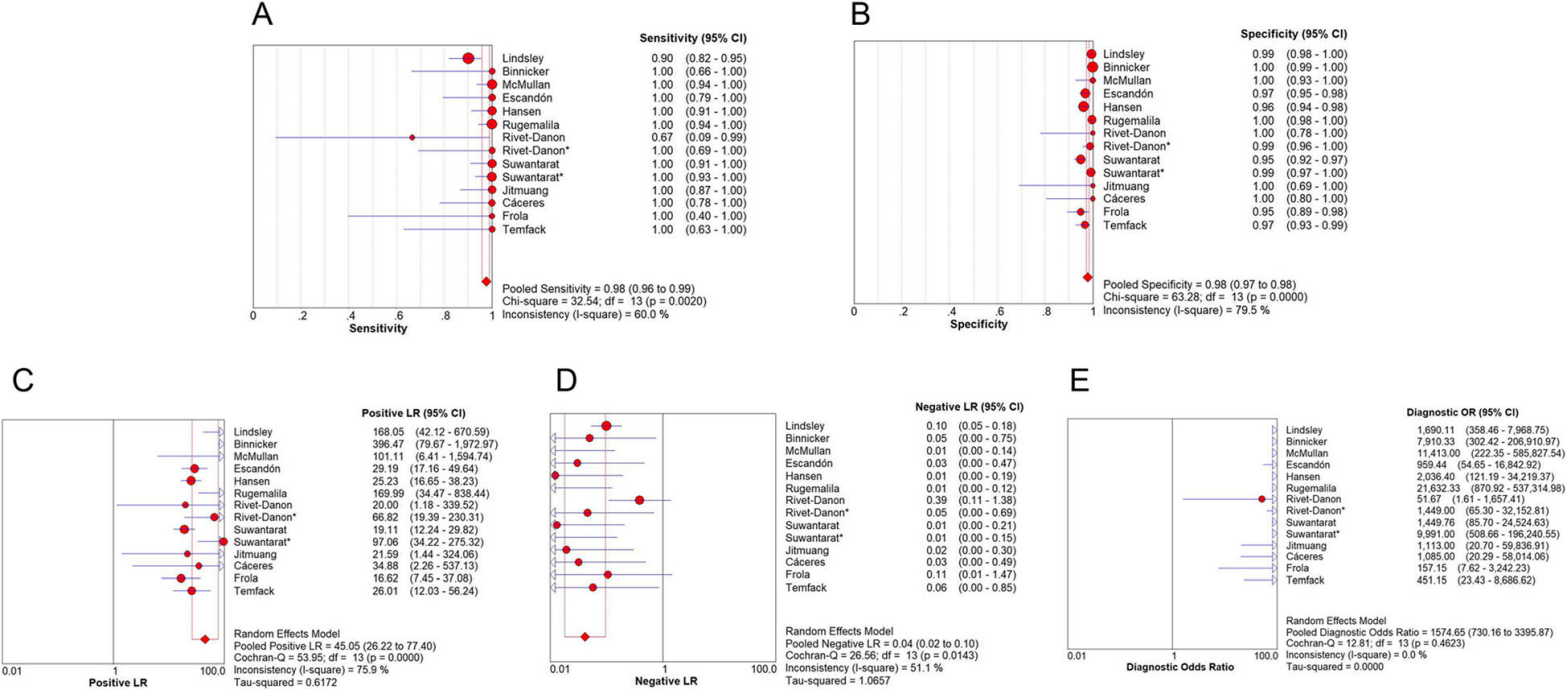
Forest plots of **a** sensitivity, **b** specificity, **c** positive LR, **d** negative LR, **e** dignostic OR of LFA for the diagnosis of cryptococcosis in serum sample

For serum specimens, the merged sensitivity and specificity values were 0.98 (95% CI: 0.96–0.99) and 0.98 (95% CI: 0.97–0.98), respectively. The average PLR of LFA in the serum was 45.05 (95% CI: 26.22–77.40) and the NLR was 0.04 (95% CI: 0.02–0.10). The merged DOR was 1574.65 (95% CI: 730.16–3395.87) and AUC was 0.9766. The results are shown in Figs. 3, 5 a.

**Fig. 4:**
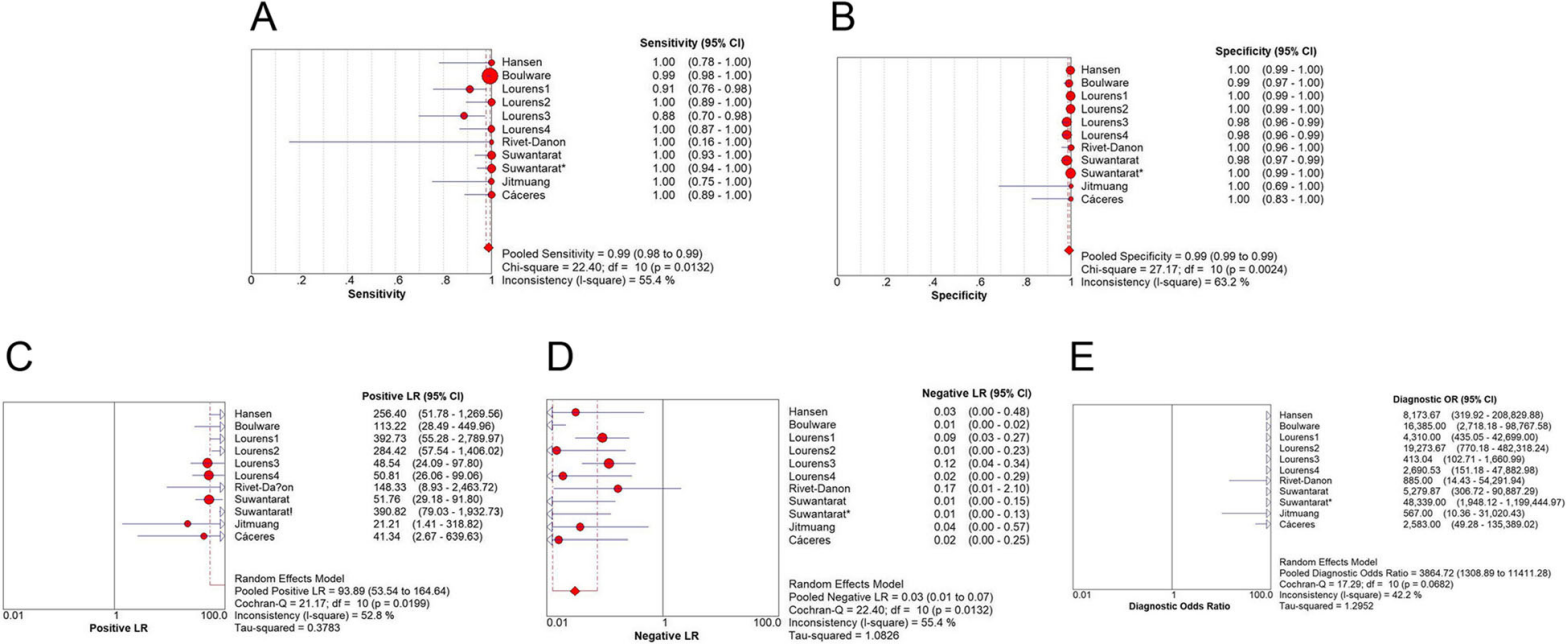
Forest plots of **a** sensitivity, **b** specificity, **c** positive LR, **d** negative LR, **e** dignostic OR of LFA for the diagnosis of cryptococcosis in CSF sample

**Fig. 5:**
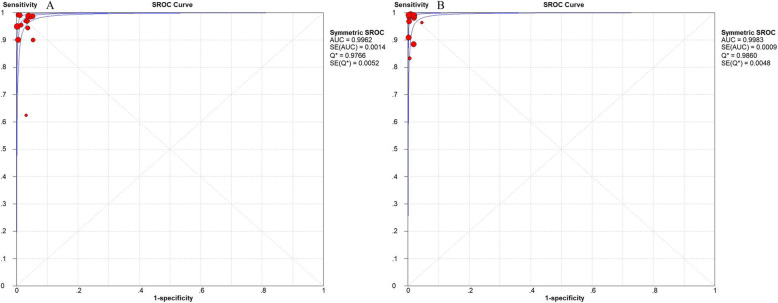
Forest plots of SROC curve of the sample in **a** serum sample and **b** CSF sample

For CSF specimens, the merged sensitivity and specificity values were 0.99 (95% CI: 0.98–0.99) and 0.99 (95% CI: 0.99 to 0.99), respectively. The average PLR of LFA in CSF was 93.89 (95% CI: 53.54–164.64) and the NLR was 0.03 (95% CI: 0.01–0.07). The merged DOR was 3864.72 (95% CI: 1308.89–11,411.28) and AUC was 0.9983. The results are shown in Figs. 4, 5 b.

**Fig. 6:**
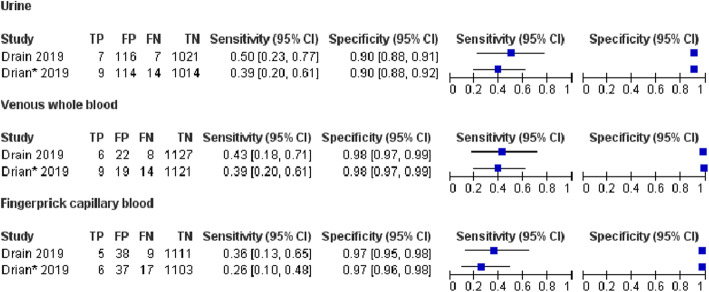
Forest plots of the sensitivity and specificity of LFA for the diagnosis of cryptococcal infection in urine and blood

For other samples, the results of sensitivity and specificity are shown in Fig. 6.

### Publication bias

**Fig. 7:**
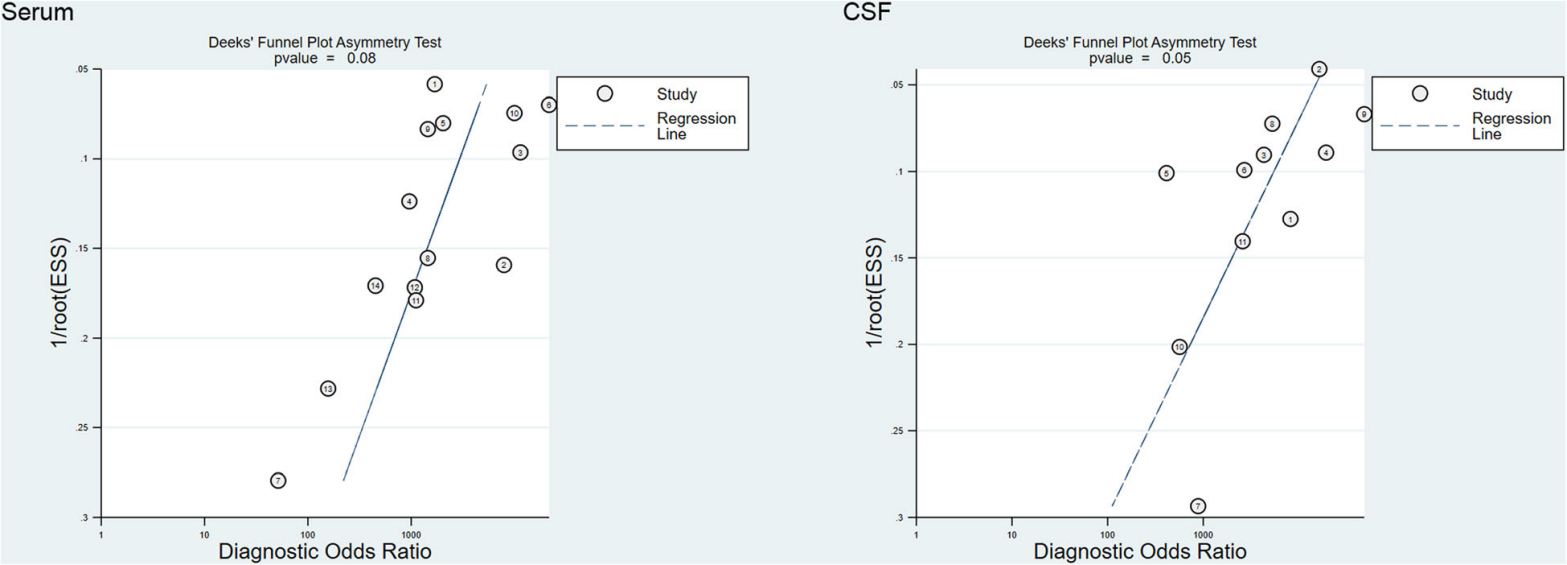
Deeks’ funnel plot asymmetry test to assess publication bias in estimates of diagnostic odds ratio for LFA detection of cryptococcal infections

In this meta-analysis, the data of serum and CSF samples were tested by Stata 12.0 for publication bias. Deek’s funnel plot asymmetry test was used to assess the potential published bias in the included studies. The results of serum and CSF samples indicated that there was no obvious publication bias (Fig. 7).

## Discussion

Studies have shown that cryptococcosis is a disease with a relatively high mortality rate. In low- and middle-income countries, especially in sub-Saharan Africa, the mortality rate is between 26% and 63% [26]. Additionally, deaths related to cryptococcal meningitis can still reach hundreds of thousands every year [8]. Therefore, a rapid diagnosis of cryptococcal infection is necessary for patients presenting appropriate clinical symptoms. A comprehensive search with stringent screening criteria retrieved 15 articles eligible for inclusion in the study. These 15 articles encompassed 3901 serum samples, 4403 CSF samples, 1125 urine samples, 1163 venous whole blood samples, and 1163 fingerprick capillary blood samples. Moreover, the brand of LFA-tests in the included studies were IMMY, which indicated that the data we extracted would not cause great heterogeneity because of manufacturers of different brands.

The results in meta-analysis showed that the combined sensitivity of LFA in serum and CSF was 0.98 (0.96–0.99) and 0.99 (0.98–0.99); specificity was 0.98 (0.97–0.98) and 0.99 (0.99–0.99); DOR was 1574.65 (730.16–3395.87) and 2509.29 (184.18–34,187.48); SROC AUC was 0.9962 and 0.9983, respectively.

Among these indexes, the PLR of the serum and CSF was > 10, while the NLR was < 0.1. The SROC AUC of the serum and CSF was close to 1. The SROC curve was close to the upper left corner, which indicated that the area under the curve was large. Both the AUCs were > 0.9, indicating that LFA had a relatively high overall diagnostic accuracy for serum and CSF. The DOR of serum and CSF was significant, indicating that the correct diagnosis is far larger than the wrong diagnosis. In conclusion, LFA has a high degree of accuracy in the diagnosis of serum and CSF.

The current analysis of these articles revealed several factors that can explain the observed heterogeneity: the differences in the reference methods in the studies; the same reference standard was not used in the study for identification; the interpretation of the results in LFA and reference methods may cause the artificial error.

Nevertheless, the current study has some limitations. Firstly, we collected all the relevant articles. However, it was difficult to ensure that no publication was missing. Secondly, we only included the articles published in the English language, which may contribute to bias. Thirdly, our study only included the articles from inception to August 2019. The difference in the reference standard might also lead to the heterogeneity of the included studies. Finally, meta-analyses of LFA for the diagnosis of cryptococcosis, only until 2015, were included. Thus, we could comprehensively analyze the accuracy of the LFA diagnosis of the cryptococcal infection.

## Conclusions

In summary, our meta-analysis indicated that LFA tested in serum and CSF has high diagnostic accuracy in the diagnosis of cryptococcal infection for high-risk patients, such as HIV-infected patients. LFA performed in urine, or other samples could be a screening tool for the early diagnosis of cryptococcal infection; however, additional studies are required for the substantiation of these results.

## Data Availability

All data generated or analyzed during this study are included in this published article.

## Abbreviations

AUC: Calculated the area under the curve
CSF: Cerebrospinal fluid
CM: Cryptococcal meningitis
DOR: Diagnostic odds ratio
FP: False positive
FN: False negative
HIV: Hunman immunodeficiency virus
LFA: Lateral flow immunochromatographic assay
NLR: Negative likelihood ratio
PLR: Positive likelihood ratio
QUADAS: Quality Assessment of Diagnostic Accuracy Study
SROC: Summary receiver operating characteristic
TP: True positive
TN: True negative
